# Symptom Attributions in Illness Anxiety Disorder

**DOI:** 10.1002/jclp.23765

**Published:** 2025-01-10

**Authors:** Monique L. Holden, Chien H. Gooi, Sophie Antognelli, Amy Joubert, Isaac Sabel, Lauren Stavropoulos, Jill M. Newby

**Affiliations:** ^1^ School of Psychology University of New South Wales Sydney NSW Australia; ^2^ Black Dog Institute University of New South Wales Sydney NSW Australia

**Keywords:** cognitive bias, cognitive processes, health anxiety, illness anxiety disorder, interpretive bias, somatoform disorders

## Abstract

**Objectives:**

A major characteristic of health anxiety is the tendency to attribute benign bodily sensations to serious illnesses. This has been supported by empirical research in non‐clinical samples, and samples of individuals diagnosed with Hypochondriasis. However, no study to date has explored symptom attribution styles of individuals with the DSM‐5 diagnosis of Illness Anxiety Disorder.

**Methods:**

Sixty‐one participants, including a clinical Illness Anxiety Disorder (*n* = 35) and healthy control (*n* = 26) sample, completed self‐report measures of health anxiety and an Attribution Task, whereby they were presented with eight common bodily sensations and asked to generate possible explanations for them.

**Results:**

Results showed that relative to healthy controls, participants with Illness Anxiety Disorder overall were more likely to make more serious, ‘catastrophic’ somatic attributions to symptoms, and less likely to generate non‐threatening normalising explanations. These results also extended to their initial attributions, conceptualised as the ‘jumping to conclusions’ bias, and as an exploratory index of flexibility, they were also found to make less attributions overall compared to healthy controls.

**Conclusions:**

Findings provide support for the cognitive behavioural theory of health anxiety, and highlight the importance of assessing and addressing symptom attributions with clients with illness anxiety disorder.

## 1 Introduction

Demonstrating concern and vigilance about one's health can be protective, allowing for the early identification and subsequent treatment of health issues. Whilst almost everyone experiences anxiety about their health to some degree, for approximately 3%–5% of the population, this occurs in excess and persists despite medical reassurance (Scarella, Boland, and Barsky 2019; Sunderland, Newby, and Andrews 2013). Excessive health anxiety has debilitating effects on an individual's personal, social, and occupational functioning (Scarella, Boland, and Barsky 2019), and has significant costs to their loved ones, the health system and society as a whole (Barsky et al. 2001; Fink, Ørnbøl, and Christensen 2010). Despite these impacts, research into health anxiety, spanning both aetiology and treatment, is limited when compared to other mental health disorders. It thus remains a priority to understand how health anxiety develops, and how it is maintained, to continue to expand and refine effective treatment options (Rachman 2012).

Health anxiety is characterised by an elevated level of worry and excessive preoccupation with one's health status (Scarella, Boland, and Barsky 2019). Being characterised by anxiety, it is thought that biased cognitions play a critical role in its development and maintenance (Eysenck 2014). Indeed, the cognitive behavioural model of health anxiety—one of the most dominant in the literature—posits that the disorder may arise due to dysfunctional negative cognitive biases and occurs when benign or commonplace bodily sensations are misinterpreted as evidence of being severely ill (Warwick and Salkovskis 1990). For example, for an individual with health anxiety, a typically neutral symptom such as a bruise may be attributed to leukaemia, as opposed to the result of bumping into something (Salkovskis, Warwick, and Deale 2003).

Numerous studies have found evidence of these negative cognitive biases in health anxiety, both in non‐clinical and to a lesser extent, clinical samples (e.g., Fergus and Valentiner 2011; Hadjistavropoulos, Craig, and Hadjistavropoulos 1998; Hadjistavropoulos et al. 2012; Hitchcock and Mathews 1992; Leonidou and Panayiotou 2018; Lorimer et al. 2020; Rief, Hiller, and Margraf 1998). Importantly, these biased cognitions have been found to result in more chronic and persistent cases of health anxiety (Barsky et al. 2000; Gautreau et al. 2015), and thus should remain a crucial intervention target. It is, however, important to note that the few clinical samples researched in these studies have relied on participants diagnosed with Hypochondriasis; a DSM‐IV disorder that is largely outdated and no longer used. DSM‐IV Hypochondriasis was replaced in the DSM‐5 with illness anxiety disorder (IAD) and somatic symptom disorder (SSD) due to its limited clinical validity and utility (Kikas et al. 2024) and the stigma associated with the label (Starcevic 2013). Thus, updated research that better reflects the current diagnostic criteria is crucial.

Studies looking at specific symptom attribution styles, that is, how individuals view the likely *cause* of their symptoms, have also been conducted. MacLeod, Haynes and Sensky (1998) were the first to investigate how individuals with elevated self‐reported health anxiety attributed the cause of various common bodily sensations. Their study aimed to see if the way that health anxious individuals thought about symptoms was specifically attributed to somatic (i.e., physical illness) reasons, as opposed to other possible explanations, such as psychological (i.e., mental health) or normalising (i.e., situational, or environmental) reasons. They found that individuals with elevated self‐reported health anxiety had a greater tendency to make somatic illness attributions to a number of imagined symptoms, whilst those with self‐reported generalised anxiety were more likely to make psychological attributions.

Weck et al. (2012) and Neng and Weck (2013) extended this study further to a clinical sample of treatment‐seeking individuals with the DSM‐IV defined Hypochondriasis and anxiety disorders, as well as healthy controls. They also further categorised the somatic attributional style into different severity levels (i.e., mild, moderate, and serious illnesses) to account for the potential difference in ‘catastrophic’, as opposed to more minor, somatic interpretations. Using The Attribution Task, whereby individuals are presented with a number of commonplace symptoms and asked to spontaneously list as many explanations that come to mind over the span of 1 minute per symptom, they found that patients with Hypochondriasis were more likely to attribute ambiguous bodily sensations to moderate or serious illnesses and provided fewer normalising explanations. This pattern of attributional style differentiated them from those with other anxiety disorders and healthy controls. Put together, the findings indicate that it is not just the misinterpretation of bodily sensations that characterise health anxiety, but rather the misattribution of these symptoms to moderate to serious, catastrophic somatic illnesses, at the exclusion of other explanations. These studies await replication.

Although the aforementioned studies have improved our understanding of health anxiety, it is important to highlight their main limitations. One common criticism is the overreliance on self‐report measures and non‐clinical samples. Moreover, all previous, albeit limited, studies on clinical levels of health anxiety have studied samples of individuals diagnosed with Hypochondriasis (e.g., Barsky et al. 2001; Neng and Weck 2013; Rief, Hiller, and Margraf 1998). The DSM‐IV Hypochondriasis diagnosis, which centred around the conviction or belief that one was seriously ill despite negative medical evidence, was criticised for being too narrow, overly restrictive, and difficult to operationalise (Creed and Barsky 2004; Fink et al. 2004). The new DSM‐5 disorders of IAD and SSD arguably allow for much broader types of worries about health and illness, and they have promisingly been found to more reliably identify individuals with clinically significant health anxiety compared to Hypochondriasis (Newby et al. 2017). Specifically, IAD in the DSM‐5 is characterised by an excessive preoccupation with having or acquiring a serious disease, high levels of anxiety about one's health, and maladaptive behaviours (e.g., body checking, avoidance, reassurance seeking) (American Psychiatric Association 2013). No study to date has examined the cognitive biases or symptom attribution styles in individuals with IAD, nor compared them to healthy controls. There have also been no studies investigating this in Australian samples, which is important to consider given that previous research has found that interpretation biases about physical sensations appears to differ by culture (Taylor and Asmundson 2004). This study thus sought to address these gaps.

Another proposed limitation within the literature is the predominant focus on the *content* of health anxious attributions, for example, by specifically looking at whether the thought content is benign or catastrophic. More recent cognitive approaches stress the importance of understanding *how* individuals think. For example, inflexibility and rigidity in one's beliefs or interpretations has been found in other mental health conditions, such as panic disorder, generalised anxiety disorder and major depressive disorder (Lee and Orsillo 2014; Nagata et al. 2018; Zhu et al. 2021). Furthermore, there are also a number of unhelpful thinking styles, such as the ‘jumping to conclusions’ bias, which have been found in other mental health conditions (Aderka et al. 2013; Everaert et al. 2018). These would be important to consider in the context of health anxiety, for example, by assessing how flexible an individual is in their interpretations, as well as the initial conclusion that one makes about certain symptom experiences.

To address these limitations, this study sought to examine and compare symptom attributions in individuals with IAD and those without IAD, by using the Attribution Task and administering a widely used self‐report measure of catastrophic cognitions. Based on previous research from individuals with elevated self‐reported health anxiety and Hypochondriasis diagnoses (e.g., Barsky et al. 2001; Neng and Weck 2013), we hypothesised that individuals with IAD would be more prone to attributing bodily sensations to moderate to serious somatic illnesses, rather than a neutral experience, compared to healthy controls. To test for flexibility in attributions as well as specific cognitive biases, such as ‘jumping to conclusions’, several additional indices were also used, including: the overall number of attributions made, the proportion interpreted as a specific attributional category in the context of the total number of attributions made, and participants' initial attributions when first presented with the different symptoms of the Attribution Task. These measures were exploratory, given it is the first time they have been researched in the health anxiety literature.

## 2 Materials and Methods

### 2.1 Participants

This study took place at the University of New South Wales. Participants included a treatment‐seeking clinical sample meeting criteria for IAD (*n* = 35) and a healthy control sample (*n* = 26) who did not meet criteria for IAD. The clinical sample was recruited between June 2017 and February 2018 as part of a previously published treatment trial by Antognelli, Sharrock and Newby (2020), which tested a computerised cognitive bias modification (CBM‐I) training programme. Participants in the clinical sample were recruited using flyers posted on the university campus and from the university's online student participant pool. The healthy control sample was recruited between January and May 2022 solely via the university's online community participant pool. All community‐based participants were paid at a rate of $20AUD/h, whilst any undergraduate students from the clinical sample were reimbursed with course credit.

Eligibility criteria across both samples included being over 18 years of age, having access to a computer/smartphone and internet, and sufficient English proficiency. Additional eligibility criteria for the clinical sample included a score on the short health anxiety inventory (SHAI) ≥ 20 (Salkovskis et al. 2002), which has been suggested as a useful clinical cut‐off score for identifying elevated levels of health anxiety (Alberts et al. 2012; Kocjan 2016). The clinical sample was also screened for acute suicidality, current risk of self‐harm, and/or current substance dependence, and were deemed ineligible to participate if these were present. Additional eligibility criteria for the healthy control sample included a score on the Short Health Anxiety Inventory < 20.

Ethics approval for the study was acquired by the University's Human Research Ethics Committee (HREC; Reference Number: 3563).

### 2.2 Measures

#### 2.2.1 Self‐Report

##### 2.2.1.1 Short Health Anxiety Inventory

The short health anxiety inventory (Salkovskis et al. 2002) is an 18‐item self‐report measure of health‐related anxieties, independent of physical health status. Items assess an individual's worry about health, awareness of bodily sensations or changes, and feared consequences of having an illness, on a four‐point Likert scale from 0 to 3. In a systematic review and meta‐analysis by Alberts et al. (2013), internal consistency for the SHAI ranged from good to excellent (α = 0.74–0.96) across 16 studies that spanned clinical and non‐clinical samples. It also demonstrated good convergent and divergent validity. Cronbach's alpha for the current study was 0.96.

##### 2.2.1.2 Cognitions About Body and Health Questionnaire

The cognitions about body and health questionnaire (CABAH; Rief, Hiller, and Margraf 1998) is a 31‐item self‐report tool used to measure cognitions about health, body, and illness. Items assess several factors, including catastrophising interpretations of bodily complaints, autonomic sensations, bodily weakness, intolerance of bodily complaints and health habits. Participants rate the extent to which each statement applies to them on a four‐point Likert scale from 0 to 3, with higher values indicating higher severity. The CABAH has been shown to have high internal consistency (α = 0.90, Rief, Hiller, and Margraf 1998; α = 0.82, Bailey and Wells 2015), as well as sensitivity to change in clinical samples and treatment studies (Liao and Huang 2021; Newby et al. 2016). Cronbach's alpha for the CABAH in the current study was 0.91.

#### 2.2.2 Diagnostic Interview

The anxiety disorders interview schedule for DSM‐5 (ADIS‐5; Brown and Barlow 2014) IAD module was administered to the clinical sample to determine diagnostic status. The ADIS‐5 is a semi‐structured interview that assesses DSM‐5 criteria for a range of anxiety and related disorders, in this case, IAD. Newby et al. (2017) found this module to have good inter‐rater reliability, with a Kappa estimate of 0.80.

#### 2.2.3 The Attribution Task

The attribution task (Neng and Weck 2013) was administered online to assess participants interpretations of common bodily symptoms. Participants were instructed to imagine that they were currently experiencing one of eight bodily sensations (e.g., a prolonged headache, dizziness, fatigue), and asked to generate as many possible explanations for these sensations, at a time of 1 minute per sensation. Both samples were unable to progress to the next sensation until 1 minute had elapsed.

Participant responses were coded into three categories: normalising (e.g., external or environmental reasons such as overexertion, dehydration), psychological (e.g., emotional distress, anxiety) and somatic (e.g., diseases such as asthma or cancer). The latter category was further graded into mild (e.g., cold/flu, sore throat), moderate (e.g., heatstroke, thyroid problems), or serious (e.g., stroke, brain tumour) diseases to account for differences in the severity of somatic attributions. Classifications were made using a coding manual, The seriousness of illness rating scale‐revised (SIRS‐S; Rosenberg et al. 1987), which had been updated by Weck, Bleichhardt and Hiller (2009). Given the original coding manual is based in German language, it was translated to English using the online translation software, Google Translate, for the current study. The coding process involved assigning each of the participant's responses to a particular attribution category. Responses were double coded by two researchers who were blind to participants' diagnostic status. Inter‐rater reliability was κ = 0.88, *p* < 0.001, 95% CI (0.86, 0.90) after the initial classification, which is considered a strong agreement (McHugh 2012), and any discrepancies were resolved using a team‐based discussion. The total number of different attributions made per participant overall was calculated, as was the total number of different attributions given as the first response across all eight symptoms. As an index of flexibility, relative frequencies were also computed (e.g., the number of serious somatic attributions a participant listed divided by the total number of attributions they had made overall).

### 2.3 Procedure

Following recruitment, applicants read information about the study, before providing electronic informed consent. The clinical sample then completed online questionnaires before their treatment trial, including a demographics questionnaire, the SHAI, and the CABAH, before completing the Attribution Task. Participants who scored ≥ 20 on the SHAI then completed a 20‐min phone diagnostic interview, using the anxiety disorders interview schedule for DSM‐5 (ADIS‐5; Brown and Barlow 2014), and were further screened for self‐harm and suicide risk, as well as substance dependence, before their allocation to treatment conditions. All diagnostic interviews were completed by either a clinical psychologist or registrar psychologist who had been trained in clinical and diagnostic assessment. The interviews were not double coded by an independent blind rater. However, past research using this tool has shown that inter‐rater reliability estimates are high between initial interviewers and independent blinded raters for the IAD diagnosis (κ = 0.92) (Newby et al. 2017).

For the control sample, participants first completed the SHAI to screen for any indications of health anxiety. Participants with elevated levels (≥ 20) were informed that they did not meet inclusion criteria for the study and were advised of a number of support services. Those who scored < 20 on the SHAI completed the demographics questionnaire, the CABAH and the Attribution Task. After this, participants then received a 5‐min phone call to complete brief screening questions to rule out the possibility of IAD, thus confirming eligibility criteria. The screening questions included, for example, ‘Over the last 6 months, have you continually feared or believed that you might have, or that you might contract a serious physical disease or illness?’ and ‘Are there actions you perform regularly to check to see whether you have developed a disease or illness?’. These screening questions were drawn from the ADIS‐5 IAD module (covering Criterion A and D from the DSM‐5) to ensure control participants did not meet criteria for IAD.

### 2.4 Statistical Methods

All analyses were conducted using SPSS version 26.0. Categorical variables were compared using chi‐square tests, whilst continuous measures were compared using independent samples *t*‐tests. To compare attribution categories and first responses between the two groups, independent samples *t*‐tests were used. Based on a priori calculations, we estimated a sample size of at least 58 was needed to detect a medium to large effect size between the two groups (Cohen's *d* = 0.75, 80% power, alpha of 0.05, two tailed). This predicted effect size was conservatively based off Neng and Weck (2013) study, noting that group differences in the types of attributions for hypochondriasis compared to healthy controls were large (*d* = 1.13–2.88).

## 3 Results

### 3.1 Participant Characteristics

Sixty‐five participants met criteria for, and participated in the study, however, four participants within the control sample were excluded from analysis due to misunderstanding the Attribution Task. These excluded participants mistakenly listed how they would cure the symptom, as opposed to focusing on the cause of the symptom. The sample included for analysis thus consisted of 61 participants. Of these participants, 35 (57.38%) met criteria for IAD and 26 (42.62%) were classified as healthy controls, without IAD. The majority of participants were female (75.41%), never married (78.61%) and of Asian descent (61.02%). Table 1 provides an overview of the sample characteristics. No differences were found between the groups on gender, marital status or ethnicity. However, the IAD group was younger, less educated and less likely to be employed than controls. Therefore, in addition to the original planned *t*‐tests, analyses of covariance (ANCOVA) were conducted to compare the groups, controlling for these sociodemographic variables (age, employment, education).

**TABLE 1:** Sample characteristics.

	IAD (*n* = 35)		Healthy controls (*n* = 26)		Total (*n* = 61)		Statistic
	M	SD	M	SD	M	SD	
Age (years)	21.51	2.90	27.12	9.53	23.90	7.10	*t*(59) = 3.29, *p *< 0.01
SHAI (total)	26.74	6.09	6.46	4.93	18.10	11.55	*t*(59) = −13.91, *p *< 0.001
CABAH (total)	49.40	10.26	28.38	9.06	40.44	14.27	*t*(59) = −8.31, *p *< 0.001
Catastrophising interpretations	21.31	6.72	11.96	4.74	17.33	7.53	*t*(59) = −6.06, *p *< 0.001
Autonomic sensations	6.94	2.33	3.38	2.23	5.43	2.88	*t*(59) = −6.02, *p *< 0.001
Bodily weakness	9.23	3.77	4.54	3.54	7.23	4.33	*t*(59) = −4.93, *p *< 0.001
Intolerance of bodily complaints	6.20	1.23	3.12	1.63	4.89	2.08	*t*(59) = −8.42, *p *< 0.001
Health habits	5.71	1.07	5.38	1.90	5.57	1.48	*t*(59) = −0.86, *p* = 0.39
	*n*	%	*n*	%	*n*	%	
Gender							χ2 (2) = 3.97, *p* = 0.14
Male	6	17.14	7	26.92	13	21.31	
Female	29	82.86	17	65.38	46	75.41	
Prefer not to answer	0	0	2	7.69	2	3.28	
Education							χ2 (5) = 21.62, *p* < 0.001
High school	23	65.71	5	19.23	28	45.90	
Tertiary—Diploma	3	8.57	0	0	3	4.92	
Tertiary—Bachelors	5	14.29	16	61.54	21	34.43	
Tertiary—Masters/Doctoral	4	11.43	3	11.54	7	11.46	
Tertiary—Professional degree	0	0	1	3.85	1	1.64	
Other	0	0	1	3.85	1	1.64	
Employment status							χ2 (4) = 12.14, *p* < 0.05
Student	29	82.86	11	42.31	40	65.57	
Part time	3	8.57	5	19.23	8	13.11	
Full time	3	8.57	7	26.92	10	16.39	
Self‐employed	0	0	2	7.69	2	3.28	
Prefer not to answer	0	0	1	3.85	1	1.64	
Marital status							χ2 (3) = 4.92, *p* = 0.18
Never married	30	85.71	18	69.23	48	78.69	
De‐facto	4	11.43	3	11.54	7	11.48	
Married	1	2.86	3	11.54	4	6.56	
Other	0	0	2	7.69	2	3.28	
Ethnicity							χ2 (3) = 2.87, *p* = 0.41
Caucasian descent	7	21.21	6	23.08	13	22.03	
Asian descent	20	60.60	16	61.54	36	61.02	
Indian descent	3	9.09	4	15.38	7	11.86	
Other	3	9.09	0	0	3	5.08	

The mean SHAI score for the IAD group was 26.74 (SD = 6.09) and indicated clinically elevated levels of health anxiety (Alberts et al. 2013). This score was significantly higher than the healthy control group, which was 6.46 (SD = 4.93) and outside of the clinical range, *t*(59) = −13.91, *p* < 0.001. On the CABAH measure, the IAD group had higher dysfunctional cognitions about illness than the healthy control group. This significant difference was also found for all subscales of the CABAH, except for the health habits subscale (see Table 1).

### 3.2 Total Attributions

Table 2 shows the means, standard deviations and confidence intervals for each group on the Attribution Task. The IAD group made fewer attributions overall on the Attribution Task than the control group.

**TABLE 2:** Means, standard deviations and statistics of the overall number of attribution categories, and total attributions, for participants with IAD and healthy controls.

	IAD (*n* = 35)		Healthy controls (*n* = 26)		Statistic	95% CI
	M	SD	M	SD		
Normalising	5.31	3.35	14.15	5.64	*t*(59) = 7.64, *p *< 0.001	6.53–11.15
Psychological	3.97	2.71	6.27	4.94	*t*(59) = 2.32, *p *< 0.05	0.32–4.28
Somatic—Mild	1.14	1.52	1.38	1.53	*t*(59) = 0.61, *p* = 0.54	−0.55–1.03
Somatic—Moderate	6.14	4.89	5.42	4.17	*t*(59) = −0.60, *p* = 0.55	−3.10–1.66
Somatic—Serious	3.34	4.78	0.50	1.27	*t*(59) = −2.95, *p *< 0.01	−4.77 to −0.91
Total attributions	19.91	10.48	27.73	11.55	*t*(59) = 2.76, *p *< 0.01	2.15–13.49

Abbreviation: CI, confidence interval.

### 3.3 Normalising Attributions

Frequent normalising attributions included ‘lack of sleep’, ‘not enough food or drink consumed’, ‘too much screen time’, and weather conditions e.g., ‘too hot’ or ‘too cold’. Participants with IAD made fewer normalising attributions than healthy controls.

### 3.4 Psychological Attributions

Typical psychological attributions made by participants included ‘stress’, ‘anxiety/nervousness’, ‘depression’ and ‘insomnia’. The IAD group made fewer psychological attributions than the control group.

### 3.5 Somatic Attributions

Examples of common somatic attributions included ‘cold’, ‘flu’, ‘indigestion’ (mild illnesses), ‘migraine’, ‘low blood pressure’, ‘COVID‐19’, ‘food poisoning’ (moderate illnesses), and ‘cancer’, ‘tumour’, ‘stroke’ or ‘Parkinson's disease’ (serious illnesses). There were no significant group differences on the frequency of mild or moderate somatic illness attributions. However, participants with IAD were found to more frequently attribute symptoms to a serious somatic illness than healthy controls.

### 3.6 First Symptom Attributions

Participant's first responses for each symptom of the attribution task were then analysed. Results indicated that relative to the healthy controls, participants with IAD were more likely to initially list a serious somatic illness, and less likely to provide a normalising attribution as their first response. No significant group differences were found for psychological, mild somatic, and moderate somatic attributions, listed as a first response. Means and standard deviations for each group's first attribution are presented in Table 3.

**TABLE 3:** Means, standard deviations and statistics of the frequency of first ordered attributions for participants with IAD and healthy controls.

	IAD (*n* = 35)		Healthy controls (*n* = 26)		Statistic	95% CI
	M	SD	M	SD		
Normalising	2.20	1.86	4.27	1.61	*t*(59) = 4.54, *p*< 0.001	1.16–2.98
Psychological	2.00	1.28	1.81	1.13	*t*(59) = −0.61, *p* = 0.55	−0.83–0.44
Somatic—Mild	0.49	0.70	0.38	0.64	*t*(59) = −0.58, *p* = 0.57	−0.45–0.25
Somatic—Moderate	2.09	1.50	1.50	1.27	*t*(59) = −1.61, *p* = 0.11	−1.32–0.14
Somatic—Serious	1.02	1.27	0.00	0.00	*t*(59) = −4.12, *p *< 0.001	−1.53 to −0.53

Abbreviation: CI, confidence interval.

### 3.7 Relative Frequencies of Symptom Attributions

**FIGURE 1:**
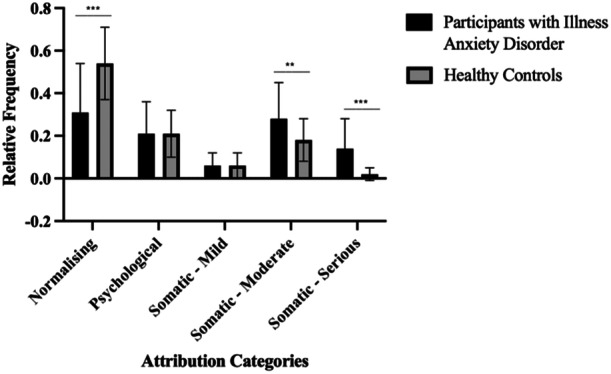
Mean relative frequencies of symptom attributions in the attribution task. *Note:* **p < 0.01; ***p < 0.001, Relative frequency = proportion of attributions in a respective category divided by total number of attributions made per participant, Error bars represent standard deviations.

Next, the number of attributions in a respective category divided by the total number of attributions made per participant was examined to determine the relative frequency, or proportion, of responses in each category (e.g., normalising, psychological, somatic). This analysis was conducted to rule out the possibility that earlier findings were due to the significant difference found in the total number of attributions made between the groups. Figure 1 shows the relative frequency of the use of different attribution categories across the two groups. In comparison to healthy controls, participants with IAD showed a higher proportion of somatic attributions that were moderate, and serious, in intensity, and lower proportion of normalising attributions. No significant group difference was found for the proportion of psychological, or mild somatic attributions.

### 3.8 Analysis of Covariance

We conducted ANCOVAs controlling for age, to compare the groups on the variables listed above (e.g., the number of total, normalising, somatic and psychological attributions), and explore whether the sociodemographic differences influenced the results. The same pattern of results as the t tests was found both with and without controlling for age, except for the total number of psychological attributions made. On this variable, once age was controlled for, there was no longer a statistically significant group difference, *F*(1, 58) = 2.60, *p* = 0.11. No changes to the pattern of results were found when controlling for employment and education status.

## 4 Discussion

This study aimed to investigate whether individuals with DSM‐5 IAD demonstrate biases in their attribution of commonly experienced, yet ambiguous somatic symptoms, such as a headache or fatigue. Compared to healthy controls, individuals with IAD generated fewer attributions to symptoms overall, and of the attributions that they did generate, a higher proportion of them were somatic illnesses that were either moderate or serious in their intensity and consequences. They were also less likely to generate normalising explanations. Expanding past research, we found these results also extended to an individual's initial attribution, in which individuals with IAD were more likely to ‘jump to’ a serious somatic attribution as their first response and less likely to consider a normalising explanation initially.

These findings are consistent with the previous literature on health anxiety (e.g., Hadjistavropoulos, Craig, and Hadjistavropoulos 1998; Hitchcock and Mathews 1992; Leonidou and Panayiotou 2018; MacLeod, Haynes, and Sensky 1998; Neng and Weck 2013; Rief, Hiller, and Margraf 1998). Importantly, however, the current study extends these findings beyond samples of self‐reported health anxiety and DSM‐IV Hypochondriasis, and is the first to show evidence of catastrophic attribution biases in individuals diagnosed with IAD. Whilst these results do need to be interpreted within the context of not controlling for other mental health or medical comorbidities in both samples, it provides interesting preliminary results on the cognitive biases in illness anxiety, and is also the first to consider indices of the ‘jumping to conclusion’ bias and provide insight into the flexibility of attributions within health anxiety.

Whilst the IAD group consistently showed fewer normalising and more serious somatic attributions than the control group across all indices in our study, we did find some minor differences in other attributional categories, when they were either analysed as an overall number of attributions, or proportion/relative frequency. When looking at overall attributions, individuals with IAD were less likely than healthy controls to make psychological attributions to symptoms, but this difference was no longer significant once age was controlled for. The healthy control sample in our study was, on average, older than the IAD sample, and research has noted that levels of stress, for example, a common psychological attribution listed in the task, typically follows an inverted U‐shaped pattern, increasing up until a certain point (typically around ones 40 s/50 s), and then decreasing thereafter (Graham and Ruiz Pozuelo 2017; Stone, Schneider, and Broderick 2017). The healthy control group, being older, may have been more likely to experience stress, therefore explaining the different sensitivities to psychological attributions found.

As expected, we found no clinical differentiation between the two groups on attributions of minor somatic illnesses, such as colds, sore throats or upset stomachs (when either analysing overall attributions or relative frequencies). However, we did find slight differences in results for moderate somatic illnesses. Similar to Neng and Weck (2013), individuals with IAD were found to have a higher *proportion* of their attributions listed as a moderate somatic illness than healthy controls, but the groups did not differ in the *total number* of moderate somatic illnesses listed. Given that on average, over half of the healthy control's attributions were normalising in nature, compared to approximately just under one‐third for the IAD sample, it makes intuitive sense that a higher *proportion* of attributions would be in the moderate somatic category for the IAD sample, despite equal numbers of these attributions overall. Nevertheless, given our relatively small sample size, the study may have also been underpowered to detect these more nuanced differences in the total number of moderate somatic attributions, and thus it should be replicated with a larger sample. Despite this, the overall results do support previous research showing the trend for symptoms to be catastrophically misinterpreted (Hadjistavropoulos, Craig, and Hadjistavropoulos 1998; Hitchcock and Mathews 1992; MacLeod, Haynes, and Sensky 1998; Neng and Weck 2013; Weck et al. 2012), specifically relating to serious somatic illnesses, and to a lesser extent, moderate illnesses.

Regarding the exploratory components of our study, in particular looking at the first attributions made by participants, we found interesting preliminary results. Individuals with IAD in this study were more likely to initially ‘jump to the conclusion’ that a symptom was a sign of serious somatic illness, as indexed by their first response, and were less likely to initially attribute symptoms to a normalising explanation compared to healthy controls. These are important findings, as research has noted that the search for explanations to phenomena is often ceased after finding one explanation that ‘fits’, and disconfirmatory evidence to this is often minimised or discounted (Everaert et al. 2018; Shaklee and Fischhoff 1982). Whilst this is the first study, to our knowledge, to explore this in individuals with IAD, it does fit with previous findings within the field, for example, studies requiring health anxious individuals to make initial judgments about health‐related scripts (Haenen et al. 2000), or complete Implicit Association Tests (Schmidt et al. 2013).

It is important to acknowledge that a limitation in our study design is that we cannot comment on how much ‘weight’ participants placed on this first attribution (*or* any attribution listed) to determine whether it was indeed their most salient conclusion drawn. Studies in the literature including an index of believability have shown interesting results. For example, Gillanders et al. (2015), tested a sample of adults with various forms of cancer, and found that threatening appraisals of cancer were related to anxiety symptoms, however, this was mediated through higher levels of cognitive ‘fusion’—the process of equating thoughts with reality, or in other words, experiencing thoughts to be highly believable and true. Future studies asking participants to rate the believability of their attributions, as in a study by Wells et al. (1995), may be helpful in determining whether the first attribution listed, which we conceptualised as the ‘jumping to conclusion’ bias, is indeed the most believable and rigidly held attribution. If this is the case, working to change an individual's initial and automatic attribution, whether this is done so more implicitly or explicitly, may be helpful in treatment.

We also found preliminary evidence that individuals with IAD provide fewer attributions overall than healthy controls when trying to explain ambiguous symptoms. There are a number of potential reasons for this; for example, it is possible that those with clinical health anxiety are more rigid and less flexible in their thinking, thus fixating on a smaller number of attributions. Inflexible or rigid interpretations are commonly found in other mental health disorders (Bronstein et al. 2022; Everaert et al. 2018; Kanai et al. 2010; Stange, Alloy, and Fresco 2017). Indeed, in a study by MacLeod et al. (1997), anxious individuals were shown to generate fewer reasons why future negative events would be unlikely to occur, and also had difficulties in generating alternative, anxiety‐reducing explanations. It may also be the case that individuals with IAD did ‘jump to the conclusion’ that the symptom was a sign of a serious illness in the first place, thus reducing their search for further explanations. However, there may be other explanations to this preliminary finding. For example, individuals with IAD may have experienced heightened state anxiety due to the imagination‐based nature of the task, thus impairing performance (Vytal et al. 2012), typing speed could have impacted the results (Forthmann et al. 2017), or the financial reimbursement paid to healthy controls could have influenced effort on the task. Replication of this study, considering these potential ideas, would be beneficial.

### 4.1 Theoretical and Clinical Implications

We should note that the data was gathered cross‐sectionally, and so we cannot definitively conclude that these cognitions *cause* anxiety/distress in individuals with IAD, and as mentioned earlier, other potential mental health or medical comorbidities that were not assessed or controlled for in this study may provide alternative explanations, at least in part, to the group differences. Nevertheless, our findings do provide support for the cognitive behavioural model put forward by Warwick and Salkovskis (1990), with individuals diagnosed clinical levels of health anxiety in our study having higher catastrophic beliefs about symptoms, and less non‐threatening normalising interpretations. Given past research has shown catastrophic thoughts about health and illness can trigger behaviours that further perpetuate health anxiety (Halldorsson and Salkovskis 2017), such as excessive internet searching for health information (Gibler et al. 2019), these cognitions continue to be a crucial target for intervention.

Importantly, our results also provide support for the use of techniques aimed at challenging and restructuring beliefs and thoughts about illness, such as those found in cognitive behaviour therapy (CBT)—an effective treatment for health anxiety (Axelsson and Hedman‐Lagerlöf 2019; Cooper et al. 2017). Specific cognitive techniques, such as providing psychoeducation to clients about the different attributional styles, helping them to generate as many other possible variations of symptom attributions (and in particular, normalising explanations, as we observed in our healthy controls), traditional ‘evidence for and against’ cognitive restructuring exercises, and survey behavioural experiments of how other healthy individuals interpret their symptoms, may all help to reduce the potency of health anxious thoughts and improve one's cognitive flexibility. Whilst there has been some research isolating the specific techniques (e.g., Kerstner et al. 2015), further research is needed to compare these techniques and identify which is the most beneficial. It would also be interesting to see whether improvements in health anxiety from cognitive, or cognitive behavioural treatment, coincides with any clinically significant changes on the Attribution Task in future studies to assess not only whether, but how, these interventions might work.

### 4.2 Limitations

Despite the strengths of this study, the results need to be interpreted within its limitations. Firstly, the samples consisted of mostly young female participants who were reimbursed for taking part in the study, which may reduce the generalisability of our findings. Secondly, as mentioned throughout, we did not assess nor control for the potential confound of other mental health or medical comorbidities, which reduces the validity of our findings being specific to IAD. Newby et al. (2017) found that 60.7% of individuals diagnosed with IAD in their sample also had another comorbid mental disorder, such as Generalised Anxiety Disorder or Major Depressive Disorder, and Hadjistavropoulos et al. (2012) additionally highlighted that health anxious cognitions differed to some extent between individuals with and without self‐reported medical conditions. It is possible that potential mental health and/or medical comorbidities (both past and present) that were not assessed in the current study could explain, at least in part, the bias detected, and thus replicating the study with this in mind is crucial. Similarly, our methodology does make it difficult to ascertain whether the control sample was indeed truly ‘healthy’. Whilst IAD was able to be confidently ruled out due to control participants not meeting Criterion A, as well as D, of the disorder, as per the DSM‐5, assessing for mental health and/or medical comorbidities would similarly be important for a more accurate and valid comparison between the two groups. Thirdl, the timing of data collection differed, with the IAD sample recruited pre‐pandemic, and the healthy controls recruited during the COVID‐19 pandemic. Research has suggested that health‐related anxieties for both clinical and non‐clinical samples escalated as a result of the pandemic (e.g., Asmundson and Taylor 2020a; 2020b; Jungmann and Witthöft 2020; Sauer, Jungmann, and Witthöft 2020), and thus it would be beneficial to replicate our findings with both samples being recruited in the same health climate. It is also important to highlight that participant's current emotional state was not assessed, for example, one's degree of state anxiety, which may have influenced the cognitive biases found (Mathews, Mackintosh, and Fulcher 1997). Future research should consider these factors as a priority.

Regarding our study design, due to the experimental nature of our research, the procedures we used did differ in several respects from the natural attribution process. Specifically, we had to rely on the imagination of bodily symptoms, as opposed to the actual experiences. Whilst this is an improvement from solely relying on self‐report measures to measure this phenomenon, such as the catastrophising subscale of the CABAH, future studies may wish to use physiological stress tasks, such as the Cold Pressor task as used in Hadjistavropoulos et al.'s (1998) study, or perhaps set daily body scans of symptoms in conjunction with a diary, as in a study by Kerstner et al. (2015), to capture and compare real time symptom attributions. In addition, whilst we did use a standardised time guide of 1 minute per symptom to replicate previous research studies (e.g., Neng and Weck 2013), it is important to recognise that this also reduces the external validity of our results. Participants either may have unusually dwelled on the symptom and its potential explanation, thus producing more attributions than they would day‐to‐day, or on the other hand, prematurely moved on to the next symptom after 1 minute, thus producing less attributions than they would in everyday life.

It is finally important to acknowledge the limitations to the coding system we used, The Seriousness of Illness Rating Scale (Rosenberg et al. 1987), revised in 2009 by Weck et al. Given that the scale was translated from German to English for the current study via the online software, Google Translate, this may have influenced validity of the coding process. Additionally, it was last revised over a decade ago, and what constitutes a mild, moderate, or serious somatic illness may change over time, particularly with advances in medicine and treatment. Not only this, but these ratings are arguably subjective in nature, for example, ‘heart disease’ being a serious somatic illness on the scale may fit one individual's perspective, but another may view this as a manageable illness. The results should thus be interpreted with this in mind, and future research may benefit from having participants not only generate symptom attributions, but also rate the perceived severity of said attributions, to account for their individual perspective.

## 5 Conclusion

This study is the first to explore how individuals with IAD, relative to healthy controls, attribute commonly experienced bodily sensations. This study had a number of strengths, including the use of a newer DSM‐5 clinical sample to replicate previous studies on Hypochondriasis, double blind coding procedures, and the testing of new indices—specifically, the ‘jumping to conclusions’ bias and flexibility of interpretations. Results showed that individuals with IAD, relative to healthy controls, generated fewer attributions to symptoms overall, and of the attributions they did generate, a higher proportion of them were somatic illnesses that were more catastrophic in their intensity and consequences. They were also less likely to generate non‐threatening normalising explanations to symptoms, with these findings additionally extending to their initial attributions. Overall, the results of this study provide useful insight into the nature of cognitions in IAD. Further research is required, however, to control for potential mental health and/or medical comorbidities between the groups, as well as to explore the causal role of these attributions, and the types of treatment techniques that may help shift this attributional style, with an emphasis on investigating whether this subsequently reduces anxiety in health anxious samples.

## Ethics Statement

Approval for this study was granted from a relevant ethical committee and informed consent was obtained from all study participants.

## Conflicts of Interest

The authors declare no conflicts of interest.

## Data Availability

The data that support the findings of this study are available from the corresponding author upon reasonable request.
